# Supplementary material for: Characterizing Physicochemical Selection in Protein Evolution with Property-Informed Models (PRIME)
Source: bioRxiv. 2026 Mar 11:2026.03.09.710461. Preprint. [Version 1] doi: 10.64898/2026.03.09.710461 (PMC13060920; doi:10.64898/2026.03.09.710461)
Supplement: Supplement 1 [file NIHPP2026.03.09.710461v1-supplement-1.pdf]

bioRxiv preprint doi: https://doi.org/10.64898/2026.03.09.710461; this version posted March 11, 2026. The copyright holder for this preprint (which was not certified by peer review) is the author/funder, who has granted bioRxiv a license to display the preprint in perpetuity. It is made available under aCC-BY 4.0 International license.

Supplementary Material

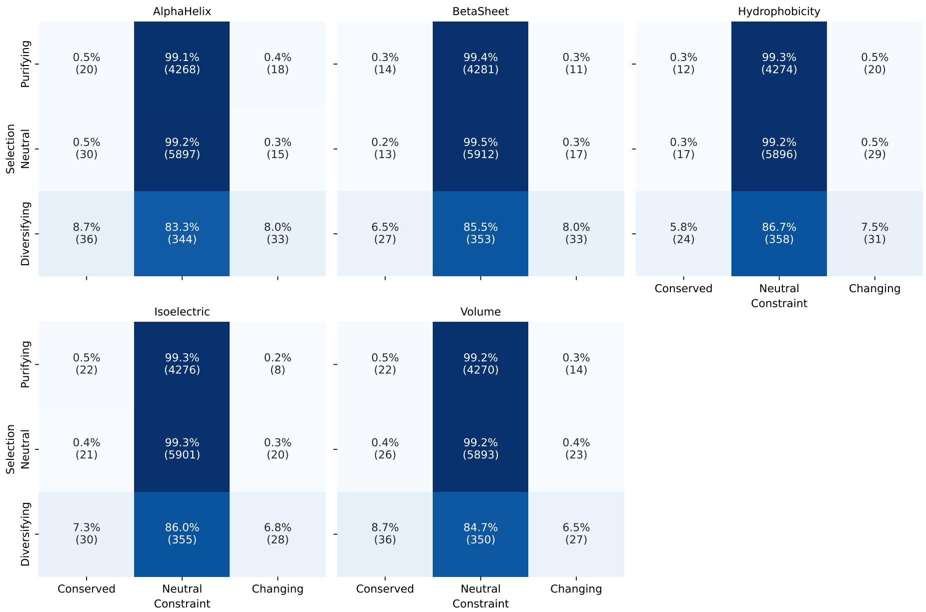

Figure S1: Conditional Probability of Physicochemical Constraints. Heatmap showing the percentage of sites within each selection class (Purifying, Neutral, Diversifying) that are Conserved, Neutral, or Changing for each physicochemical property. Raw site counts are shown in parentheses. Cryptic conservation is highlighted in the Neutral selection row, where 0.4% of sites exhibit significant property conservation despite lacking a rate-based signature of selection ($dN/dS \approx 1$).

bioRxiv preprint doi: https://doi.org/10.64898/2026.03.09.710461; this version posted March 11, 2026. The copyright holder for this preprint (which was not certified by peer review) is the author/funder, who has granted bioRxiv a license to display the preprint in perpetuity. It is made available under aCC-BY 4.0 International license.

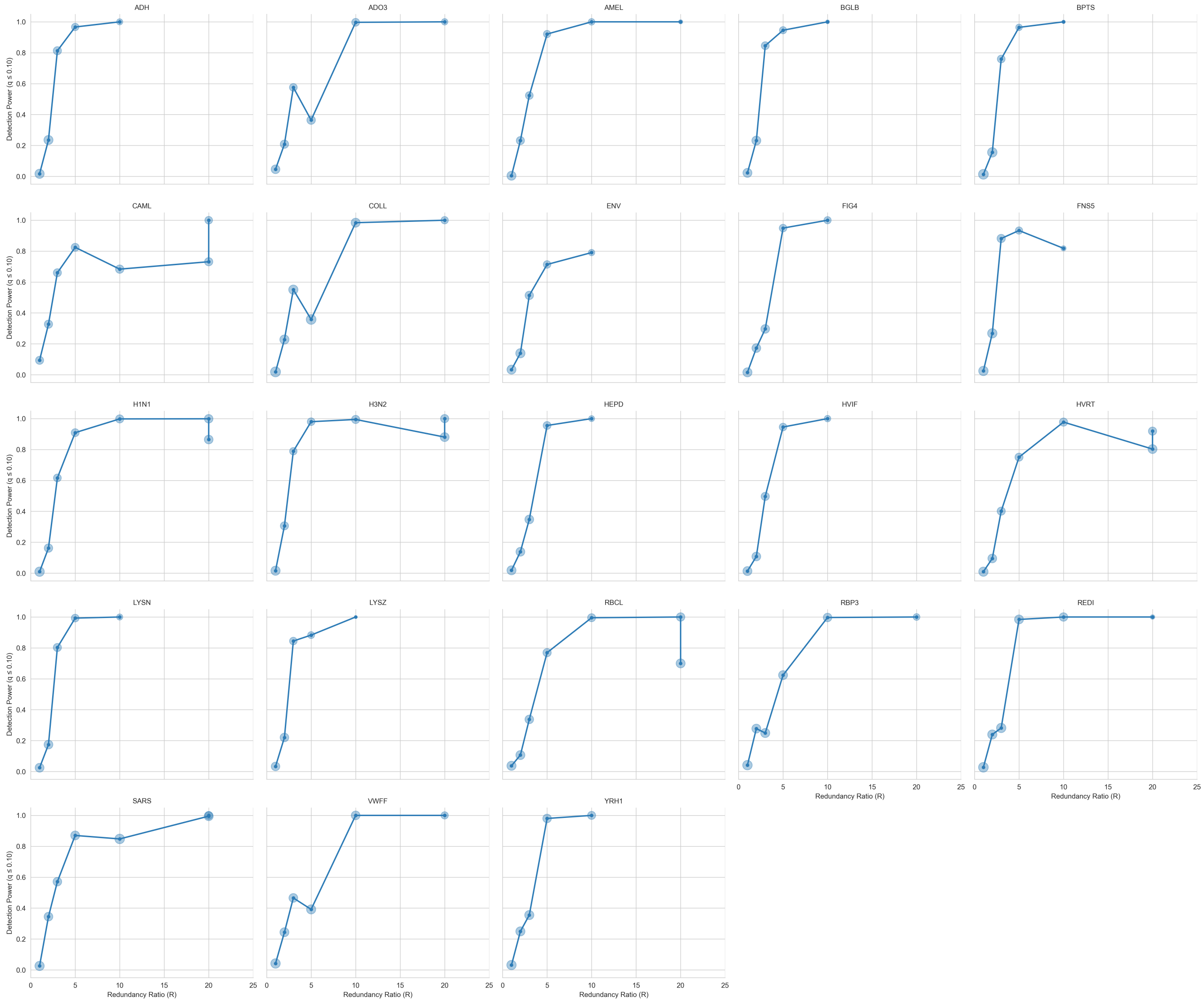

Figure S2: Detection power as a function of the redundancy ratio (R) for each of the 23 benchmark datasets. Detection is defined by the Benjamini-Hochberg corrected omnibus test ($q \leq 0.10$). Bubbles denote the relative number of simulation sites in each R bin. The consistent non-linear scaling across all genes confirms that informational depth is the primary driver of sensitivity, regardless of taxonomic origin or structural context.

bioRxiv preprint doi: https://doi.org/10.64898/2026.03.09.710461; this version posted March 11, 2026. The copyright holder for this preprint (which was not certified by peer review) is the author/funder, who has granted bioRxiv a license to display the preprint in perpetuity. It is made available under aCC-BY 4.0 International license.

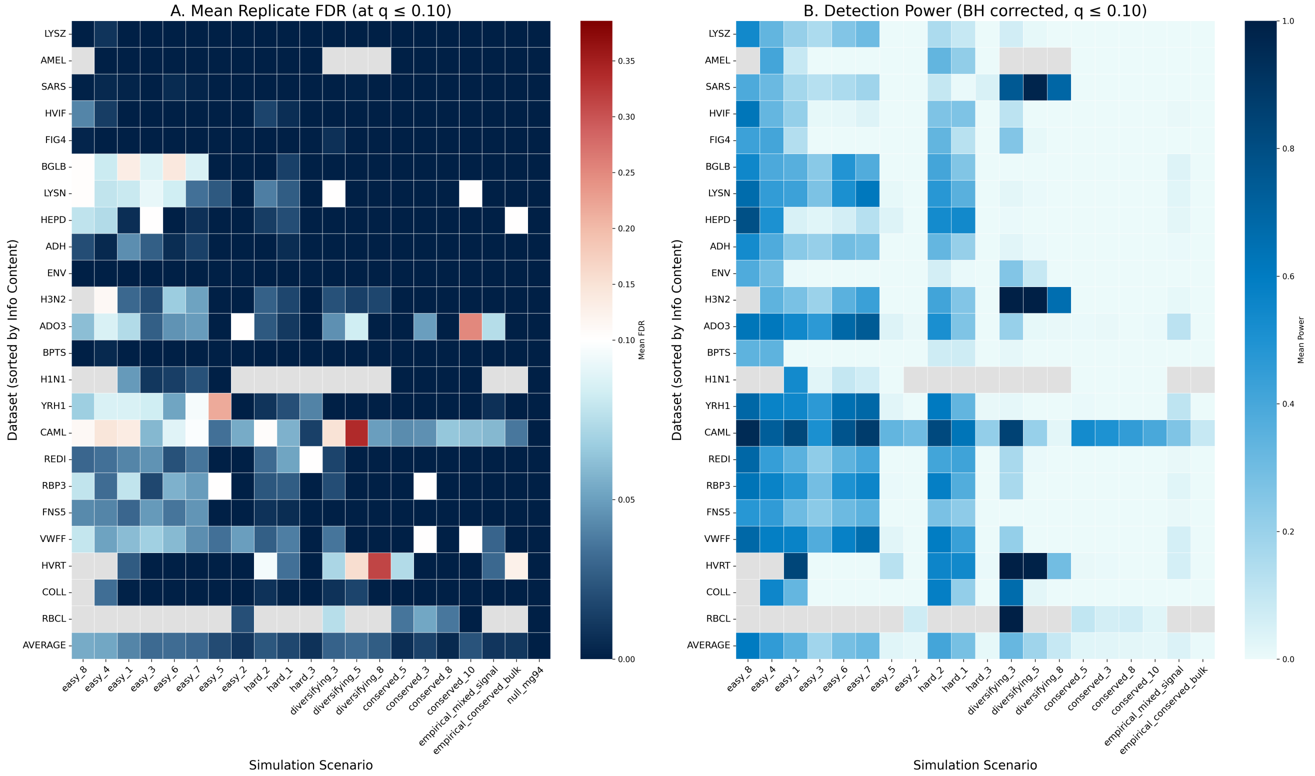

Figure S3: Dataset and scenario-specific performance S-PRIME performance on simulated data. (A) Empirical False Discovery Rate (FDR) calculated as the mean of replicate-level FDRs ($\frac{FP}{FP+TP}$) for each gene-scenario combination. The color scale is anchored at the nominal target $q \leq 0.10$ (Vik palette). (B) Mean detection power after Benjamini-Hochberg correction. The Y-axis is sorted by informational content ($L \times T$), ranging from minimal signal (LYSZ) to maximal signal (RBCL). Gray cells denote incomplete data (≤ 7 replicates). The final row (average) provides the scenario-wide mean across all benchmark genes.

bioRxiv preprint doi: https://doi.org/10.64898/2026.03.09.710461; this version posted March 11, 2026. The copyright holder for this preprint (which was not certified by peer review) is the author/funder, who has granted bioRxiv a license to display the preprint in perpetuity. It is made available under aCC-BY 4.0 International license.

Table S1: Functional and structural characteristics of the benchmark datasets.

Dataset	Function	Structural Features	Evolutionary Expectations
ADORA3 (Mammals) (Rodrigue et al., 2021)	G-protein coupled receptor	7-transmembrane alpha-helices	Conservation of core hydrophobicity for membrane insertion; variable loops
Avian REDIC1 (Jarvis et al., 2014)	NADH dehydrogenase subunit	Metabolic enzyme	Purifying selection on catalytic residues
Camelid VHH (Nguyen et al., 2002)	Single-domain antibody	Immunoglobulin fold (all-beta sandwich)	Conservation of beta-sheet propensity; diversifying selection on loops (CDRs)
Drosophila adh (Yang et al., 2000b)	Alcohol dehydrogenase	Alpha/Beta Rossmann fold	Conservation of catalytic triad and nucleotide binding domain
Encephalitis env (Yang et al., 2000b)	Viral envelope protein	Class II fusion protein (beta-sheet rich)	Surface exposure drives immune evasion; membrane fusion imposes structural constraints
Flavivirus NS5 (Kuno et al., 1998)	RNA-dependent RNA polymerase	Finger-palm-thumb domain architecture	Strict conservation of catalytic core
Hepatitis D Ag (Anisimova and Yang, 2004)	Viral capsid / RNA binding	Coiled-coil dimerization domain	Positive charge required for RNA interaction
HIV rt (Seoighe et al., 2007)	Reverse transcriptase	Heterodimer; mixed alpha/beta	Drug resistance mutations; conservation of polymerase active site
HIV vif (Yang et al., 2000b)	Viral infectivity factor	Ubiquitin ligase recruitment	Protein-protein interaction interfaces
IAV H1N1 HA (Tamuri and Dos Reis, 2022)	Viral hemagglutinin	Class I fusion (alpha-helical stem)	Stem region highly conserved (helix); head region variable (immune escape)
IAV H3N2 HA (Bush et al., 1999)	Viral hemagglutinin	Class I fusion (alpha-helical stem)	Stem region highly conserved (helix); head region variable (immune escape)
Mam. β-globin (Yang et al., 2000b)	Oxygen transport	Globin fold (all-alpha)	Strict requirement for alpha-helix propensity; heme binding pocket
Mammalian AMELX (Randall et al., 2022)	Tooth enamel formation	Intrinsically disordered protein (IDP)	Conservation of properties mediating biomineralization and nanosphere assembly
Mammalian Collagen (Mu et al., 2021)	Structural protein (Type I)	Triple helix (Gly-X-Y repeats)	Extreme conservation of volume (Gly requirement) and secondary structure propensity
Mammalian COXI	Cytochrome c oxidase subunit I	12-transmembrane alpha-helices	Conservation of hydrophobicity in TM domains; mitochondrial membrane insertion
Mammalian RBP3 (Rodrigue et al., 2021)	Retinol binding protein	Lipocalin fold (beta-barrel)	Hydrophobic pocket for retinol binding; conservation of beta-strands
Mammalian VWF (Rodrigue et al., 2021)	Blood coagulation factor	Multidomain glycoprotein	Shear-stress sensing; interaction with collagen and platelets
Primate Lysozyme (Yang, 1998)	Antibacterial enzyme	Alpha+Beta fold	Adaptive evolution in foregut fermenters (stability at low pH)
rbcL (Plant RuBisCO) (Tamuri and Dos Reis, 2022)	Carbon fixation	Alpha/Beta barrel (TIM barrel)	Extreme conservation of catalytic core; thermal stability constraints
SARS-CoV-2 S (Martin et al., 2021)	Viral spike glycoprotein	Class I fusion (alpha-helical coiled-coil)	Receptor binding domain (RBD) evolution; fusion machinery conservation
Sperm lysin (Yang et al., 2000a)	Gamete recognition/lysis	Four-helix bundle	Rapid evolution due to sexual conflict; conservation of helical structure
Streptococcus PTS (Dunn et al., 2019)	Sugar transporter	Membrane permease	Transmembrane helices
SWS1 Opsin (Bats) (Wertheim et al., 2015)	UV-sensitive opsin	7-transmembrane alpha-helices	Spectral tuning via amino acid shifts
Vertebrate Rhodopsin (Yokoyama et al., 2008)	Dim-light vision GPCR	7-transmembrane alpha-helices	Spectral tuning via electrostatic environment (charge)

bioRxiv preprint doi: https://doi.org/10.64898/2026.03.09.710461; this version posted March 11, 2026. The copyright holder for this preprint (which was not certified by peer review) is the author/funder, who has granted bioRxiv a license to display the preprint in perpetuity. It is made available under aCC-BY 4.0 International license.

Table S2: Model fit comparison and dataset characteristics for 24 benchmark alignments. For each dataset, we report the number of sequences (N), the number of codons (L), the total tree length (T, expected substitutions per site), and the PDB ID used for structural analysis (if available, with the number of mapped residues in parentheses). Datasets are sorted by the product of sequence length and tree length ($L \times T$), a proxy for total evolutionary information. Fit improvement is shown as ΔAIC relative to the $MG94 \times REV$ baseline (higher values indicate better fit). The best-fitting model for each dataset is marked with an asterisk (*) and highlighted in bold.

Dataset	N	L	T	PDB	ΔAIC(2-prop)	ΔAIC(3-prop)	ΔAIC(4-prop)	ΔAIC(5-prop)	ΔAIC(Atchley composite prop set)	CoRa ΔAIC(5-prop)
Primate Lysozyme	19	130	0.23	1LZ1 (130)	23.96*	23.07	22.46	23.64	8.21	7.24
Mammalian AMELX	44	219	0.56	*None*	10.69	21.15*	20.33	18.30	9.35	-1.70
SARS-CoV-2 S	180	1284	0.12	6VXX (972)	0.58	19.01*	18.43	16.71	8.06	1.76
HIV vif	29	192	0.91	4N9F (180)	16.65	15.43	14.98	35.97*	24.12	-1.50
SWS1 Opsin (Bats)	33	286	1.11	1U19 (280)	3.73	2.38	5.67	5.93	8.16*	3.18
Mam. β-globin	17	144	2.25	2HHB (144)	106.53	151.90	185.79	214.95*	151.25	25.60
Sperm lysin	25	134	2.54	2LIS (131)	52.20	52.54	57.02	57.51*	39.92	11.46
Hepatitis D Ag	33	196	1.77	1A92 (49)	151.98	255.32	257.84	282.17*	217.16	11.34
Drosophila adh	23	254	1.37	1A4U (254)	63.93	63.74	66.07	72.84*	45.55	9.28
Encephalitis env	23	500	0.83	1SVB (389)	5.63	7.24	12.07*	10.26	9.19	3.70
IAV H3N2 HA	349	329	1.41	4FNK (316)	4.63	3.16	4.32	3.47	15.05*	-2.00
ADORA3 (Mammals)	67	107	4.44	8X16 (95)	97.11	102.59	103.07*	102.43	73.74	1.53
Streptococcus PTS	16	639	1.76	3QNQ (409)	140.94*	140.18	138.22	136.23	73.25	16.71
IAV H1N1 HA	466	589	1.96	1RU7 (321)	146.31	146.16	144.24	149.00*	65.10	62.76
Vertebrate Rhodopsin	38	330	3.76	1U19 (327)	329.29	542.01	542.63	557.31*	358.36	6.18
Camelid VHH	212	96	14.13	1MEL (95)	95.87	125.87	123.80	135.89	186.56*	34.75
Avian REDIC1	38	653	2.45	*None*	10.32	14.10	13.20	17.87*	-3.90	-1.10
Mammalian RBP3	54	412	4.56	7JTI (158)	300.71	299.71	317.62	372.40*	138.70	54.17
Flavivirus NS5	18	342	5.56	2J7U (328)	136.16	143.78	146.18	155.73*	75.99	18.63
Mammalian VWF	62	392	5.16	1AUQ (376)	251.04	316.61	319.16	325.93*	169.69	49.14
HIV rt	476	335	6.42	1RTD (327)	362.45	370.72	390.12	434.47*	133.03	0.35
Mammalian COXI	21	510	5.08	5Z62 (506)	64.80	126.75*	125.91	123.88	50.44	-0.61
Mammalian Collagen	58	1459	2.08	5K31 (241)	703.07	984.71	988.94	992.82*	610.17	85.20
rbcL (Plant RuBisCO)	483	466	10.98	1RXO (453)	884.60	1327.37	1325.36	1327.72*	578.73	84.60

bioRxiv preprint doi: https://doi.org/10.64898/2026.03.09.710461; this version posted March 11, 2026. The copyright holder for this preprint (which was not certified by peer review) is the author/funder, who has granted bioRxiv a license to display the preprint in perpetuity. It is made available under aCC-BY 4.0 International license.

Table S3: Estimated property importance weights (λ) for the 5-prop PRIME model across 24 benchmark alignments. Datasets are sorted by the product of sequence length and tree length ($L \times T$). λs statistically significantly different from zero (Likelihood Ratio Test) are bolded and colored (Blue for conserved, Red for diversifying). Significance levels: * $p < 0.05$, ** $p < 0.01$, *** $p < 0.001$.

Dataset	Hydrophobicity	Volume	Isoelectric Point	Alpha-Helix	Beta-Sheet
Primate Lysozyme	0.55	0.94**	-0.22	0.19	0.59
Mammalian AMELX	0.13***	0.35***	0.37***	0.12	0.01***
SARS-CoV-2 S	0.19*	-0.05	-0.23***	0.09	0.04
HIV vif	0.07	0.25**	-0.04	0.02	0.51
SWS1 Opsin (Bats)	0.04	0.12	-0.02	0.15	0.15
Mam. β-globin	0.73***	0.53***	0.45***	-0.48***	0.66
Sperm lysin	0.21**	0.39***	0.07	-0.17*	0.13
Hepatitis D Ag	0.35***	0.43***	0.45***	-0.17**	0.50
Drosophila adh	0.56***	0.21	0.07	0.21*	0.38
Encephalitis env	0.57**	0.15	0.20*	-0.32*	-0.07
IAV H3N2 HA	0.03	0.11*	-0.03	0.08	0.07
ADORA3 (Mammals)	0.51***	0.26***	0.12*	0.09	0.10
Streptococcus PTS	0.43***	0.72***	-0.06	-0.02	-0.01
IAV H1N1 HA	0.16***	0.34***	-0.03	-0.01	0.12
Vertebrate Rhodopsin	0.68***	0.42***	0.85***	-0.08	0.26
Camelid VHH	0.16***	0.13***	0.14***	0.00	0.14
Avian REDIC1	-0.12***	0.08*	0.05*	-0.04	0.09
Mammalian RBP3	0.59***	0.20***	-0.02	-0.20***	0.32
Flavivirus NS5	0.22**	0.72***	0.16**	-0.16*	0.28
Mammalian VWF	0.43***	0.27***	0.19***	-0.09**	0.11
HIV rt	0.20***	0.72***	0.06**	-0.21***	0.34
Mammalian COXI	0.10	0.83***	0.74***	0.09	0.01
Mammalian Collagen	-0.01	1.20***	0.53***	-0.09**	-0.10*
rbcL (Plant RuBisCO)	0.02	0.58***	0.35***	0.01	0.05

bioRxiv preprint doi: https://doi.org/10.64898/2026.03.09.710461; this version posted March 11, 2026. The copyright holder for this preprint (which was not certified by peer review) is the author/funder, who has granted bioRxiv a license to display the preprint in perpetuity. It is made available under aCC-BY 4.0 International license.

Table S4: E-PRIME (5-prop) Parameter Estimates. For each dataset, we report the estimated property importance weights (λ) for the two mixture components. The weight of each component is shown in the second column. Values significantly different from zero (LRT $p < 0.05$) are shown in bold. Values at the upper bound of 10.00 are indicated with a dagger symbol.

Dataset	Weight	Hydrophobicity	Volume	Isoelectric Pt	Alpha Helix	Beta Sheet
Primate Lysozyme	0.80	-0.19	-3.05	-2.85	7.61	10.00†
	0.20	0.95	1.38	0.07	-0.01	0.23
SARS-CoV-2 S	0.97	10.00†	-5.54	-3.60	2.78	7.65
	0.03	0.10	0.00	-0.15	0.12	0.02
HIV vif	0.93	0.95	0.59	0.20	-0.22	0.92
	0.07	-0.36	-0.07	-0.20	0.09	0.18
SWS1 Opsin (Bats)	0.79	2.18	8.31	-0.18	10.00†	1.47
	0.21	-0.03	0.04	-0.03	-0.06	0.05
Mam. β-globin	0.91	1.36	0.95	0.18	0.14	0.22
	0.09	0.67	0.43	1.26	-0.88	1.88
Sperm lysin	0.80	9.31	-4.29	10.00†	10.00†	9.70
	0.20	0.34	0.56	0.11	-0.56	0.25
Hepatitis D Ag	0.90	3.48	4.25	2.83	-1.92	1.02
	0.10	0.31	0.21	0.55	0.22	0.50
Drosophila adh	0.94	10.00†	1.69	10.00†	-0.10	0.76
	0.06	0.62	0.34	0.05	0.21	0.64
Encephalitis env	0.62	5.11	10.00†	5.58	-1.15	2.02
	0.38	0.61	-0.02	0.15	-0.30	-0.13
IAV H3N2 HA	0.96	0.72	0.32	-0.73	-0.94	2.06
	0.04	-0.31	0.26	0.42	0.56	-0.26
ADORA3 (Mammals)	0.71	9.00	0.74	-0.04	-3.48	6.88
	0.29	0.35	0.40	0.11	0.42	-0.12
Streptococcus PTS	0.87	10.00†	0.40	0.61	-0.20	-0.41
	0.13	0.60	1.37	-0.12	-0.14	0.11
IAV H1N1 HA	0.87	-0.78	1.36	7.74	2.03	-1.24
	0.13	0.12	0.38	-0.10	-0.09	0.21
Vertebrate Rhodopsin	0.96	1.55	2.06	8.63	-0.21	1.18
	0.04	1.19	0.48	0.83	-0.52	0.05
Camelid VHH	0.95	0.35	0.24	0.13	-0.08	0.11
	0.05	-0.16	-0.06	0.22	0.05	0.42
Avian REDIC1	0.52	-0.12	0.07	0.07	-0.06	0.08
	0.48	8.85	10.00†	-5.10	6.51	7.17
Mammalian RBP3	0.82	2.04	0.64	0.83	-1.56	-0.75
	0.18	0.37	0.03	-0.28	0.57	0.89
Flavivirus NS5	0.90	-0.52	8.18	10.00†	10.00†	7.22
	0.10	0.49	1.62	0.25	-0.77	0.30
Mammalian VWF	0.73	8.89	1.50	10.00†	0.29	-0.60
	0.27	0.40	0.33	0.16	-0.15	0.12
HIV rt	0.96	10.00†	-0.54	-0.55	-4.84	2.34
	0.04	-0.03	1.12	0.17	0.00	0.21
rbcL (Plant RuBisCO)	0.97	-1.74	7.90	10.00†	-0.70	-0.33
	0.03	0.28	0.29	0.31	0.03	-0.04

bioRxiv preprint doi: https://doi.org/10.64898/2026.03.09.710461; this version posted March 11, 2026. The copyright holder for this preprint (which was not certified by peer review) is the author/funder, who has granted bioRxiv a license to display the preprint in perpetuity. It is made available under aCC-BY 4.0 International license.

Table S5: Selective Scenarios for S-PRIME Power Analysis. For each scenario, we report the biophysical importance factors (λ) applied to the 20% alternative partition. Properties: H: Hydrophobicity, V: Volume, P: Isoelectric Point (pI), α: Alpha-Helix, β: Beta-Sheet.

ID	Description	λ_H	λ_V	λ_P	λ_α	λ_β
easy_8	Easy: hydro++ vol+ pol– helix- sheet–	12.38	0.86	-5.02	-0.86	-2.37
easy_4	Easy: hydro– vol++ pol++ helix– sheet++	-4.91	3.85	1.16	-2.37	12.42
easy_1	Easy: hydro– vol++ pol- helix++ sheet–	-1.05	2.20	-0.66	11.37	-3.50
easy_3	Easy: hydro++ vol– pol++ helix++ sheet–	14.34	-1.64	9.52	2.18	-2.89
easy_6	Easy: hydro++ vol++ pol++ helix– sheet-	2.21	13.22	2.96	-4.16	-0.35
easy_7	Easy: hydro++ vol– pol++ helix- sheet++	4.94	-6.95	1.79	-0.60	13.80
easy_5	Easy: hydro– vol++ pol++ helix+ sheet-	-1.61	1.98	13.23	0.83	-0.87
easy_2	Easy: hydro+ pol+ helix- sheet+	0.95	0.09	0.63	-0.38	0.25
hard_2	Hard: hydro+ vol+ pol+ helix- sheet+	0.55	1.19	5.73	-2.56	1.07
hard_1	Hard: hydro+ vol+ pol- helix- sheet-	2.99	3.44	-1.69	-0.58	-0.99
hard_3	Hard: vol- pol+ helix+ sheet+	0.15	-0.55	0.54	1.00	0.40
diversifying_3	Diversifying: lambda=-3.0	0.00	-3.00	0.00	0.00	0.00
diversifying_5	Diversifying: lambda=-5.0	0.00	-5.00	0.00	0.00	0.00
diversifying_8	Diversifying: lambda=-8.0	0.00	-8.00	0.00	0.00	0.00
conserved_5	Conservation: lambda=5.0	0.00	5.00	0.00	0.00	0.00
conserved_3	Conservation: lambda=3.0	0.00	3.00	0.00	0.00	0.00
conserved_8	Conservation: lambda=8.0	0.00	8.00	0.00	0.00	0.00
conserved_10	Conservation: lambda=10.0	0.00	10.00	0.00	0.00	0.00
empirical_mixed_signal	Empirical Cluster 4: Strong Sheet conservation, moderate Vol diversification	-1.26	-1.99	3.41	0.27	9.39
empirical_conserved_bulk	Empirical Cluster 1: Moderate conservation on multiple props	2.99	2.93	2.25	-0.14	-0.96
null_mg94	Strict Null: All lambdas = 0 (MG94)	0.00	0.00	0.00	0.00	0.00

bioRxiv preprint doi: https://doi.org/10.64898/2026.03.09.710461; this version posted March 11, 2026. The copyright holder for this preprint (which was not certified by peer review) is the author/funder, who has granted bioRxiv a license to display the preprint in perpetuity. It is made available under aCC-BY 4.0 International license.

Table S6: S-PRIME Simulation Results by selective scenario. We report the mean False Positive Rate (FPR) and Power (sensitivity) averaged over all replicates (N) for both raw ($\alpha = 0.05$) and Benjamini-Hochberg corrected ($q \leq 0.10$) omnibus tests. For the `null_mg94` scenario, where all sites are neutral, results from both partitions are pooled into a single FPR estimate.

Scenario	N	Raw FPR	Raw Power	BH FPR	BH Power
easy_8	195	0.035	0.646	0.011	0.603
easy_4	210	0.033	0.493	0.008	0.456
easy_1	220	0.027	0.451	0.006	0.349
easy_3	221	0.023	0.216	0.003	0.183
easy_6	221	0.027	0.328	0.005	0.289
easy_7	219	0.024	0.354	0.005	0.315
easy_5	223	0.022	0.093	0.000	0.031
easy_2	222	0.015	0.064	0.000	0.017
hard_2	216	0.018	0.480	0.004	0.408
hard_1	216	0.017	0.362	0.002	0.284
hard_3	220	0.014	0.057	0.000	0.014
diversifying_3	217	0.023	0.508	0.006	0.322
diversifying_5	213	0.026	0.308	0.006	0.187
diversifying_8	211	0.029	0.194	0.005	0.091
conserved_5	226	0.015	0.082	0.000	0.029
conserved_3	226	0.016	0.082	0.000	0.025
conserved_8	226	0.015	0.064	0.000	0.023
conserved_10	226	0.015	0.055	0.001	0.019
empirical_mixed_signal	221	0.014	0.075	0.000	0.039
empirical_conserved_bulk	219	0.016	0.023	0.000	0.005
null_mg94	228	0.019	—	0.000	—

bioRxiv preprint doi: https://doi.org/10.64898/2026.03.09.710461; this version posted March 11, 2026. The copyright holder for this preprint (which was not certified by peer review) is the author/funder, who has granted bioRxiv a license to display the preprint in perpetuity. It is made available under aCC-BY 4.0 International license.

Table S7: Detailed S-PRIME Performance and Descriptive Statistics by Dataset for Scenario `easy_1` ($\lambda \approx 11$). $R = N_{subs}/N_{aa}$ denotes the Redundancy Ratio. FPR and Power are reported for raw ($\alpha = 0.05$) and BH-corrected ($q \leq 0.10$) tests.

Dataset	N	FPR (Raw)	FPR (BH)	Power (Raw)	Power (BH)	Med R_{null}	Med R_{alt}	Med S_{null}	Med S_{alt}
AMELX	10	0.002	0.000	0.286	0.091	0.5	1.4	1	8
Bacterial_PTS_trehalose_transporter_suIII	10	0.002	0.000	0.107	0.002	0.5	0.8	1	4
COL1A1	10	0.005	0.000	0.478	0.329	0.5	1.7	1	9
ENCenv	10	0.000	0.000	0.126	0.005	0.0	1.0	0	3
Fig4E	10	0.001	0.000	0.302	0.138	0.5	1.3	1	6
HIV_RT	10	0.022	0.006	0.885	0.834	1.3	5.1	6	48
HIVvif	10	0.007	0.000	0.395	0.213	0.7	1.5	2	8
HepatitisD	10	0.018	0.001	0.287	0.048	0.8	1.9	3	19
IAV-human-H1N1-HA	8	0.031	0.006	0.604	0.538	1.0	2.5	3	16
InfluenzaA	8	0.032	0.003	0.335	0.280	1.0	0.5	3	1
REDIC1	10	0.029	0.004	0.438	0.352	1.0	1.0	4	2
SARS-CoV-2-spike	9	0.003	0.000	0.177	0.172	0.0	0.0	0	0
adh	10	0.037	0.003	0.325	0.239	0.8	0.5	3	1
adora3	10	0.046	0.012	0.605	0.545	1.7	1.3	11	4
bglobin	10	0.070	0.015	0.514	0.362	1.1	1.2	7	3
camelid	10	0.072	0.037	0.830	0.825	3.7	6.6	41	26
flavNS5	10	0.036	0.003	0.428	0.307	1.0	1.0	4	2
lysin	10	0.043	0.009	0.559	0.430	1.2	1.2	7	4
lysozyme	10	0.006	0.000	0.265	0.208	0.5	0.5	1	1
rbcL	5	0.050	0.015	0.726	0.698	3.0	3.1	24	12
rbp3	10	0.040	0.010	0.559	0.482	1.4	1.3	8	4
vwf	10	0.042	0.010	0.618	0.567	1.6	1.5	10	5
yokoyama.rh1.cds.mod.1-990	10	0.046	0.013	0.639	0.548	1.4	1.5	10	5

bioRxiv preprint doi: https://doi.org/10.64898/2026.03.09.710461; this version posted March 11, 2026. The copyright holder for this preprint (which was not certified by peer review) is the author/funder, who has granted bioRxiv a license to display the preprint in perpetuity. It is made available under aCC-BY 4.0 International license.

Table S8: Site Classification Counts across Benchmark Datasets. Sites were classified based on the decision tree in Figure 6, integrating MEME selection detection ($p \leq 0.05$) with PRIME physicochemical structure (FDR ≤ 0.10). *Adaptive* classes (left) correspond to sites under diversifying selection; *Neutral/Purifying* classes (right) correspond to sites without rate elevation. Abbreviations: Constr. (Constrained), Div. (Diversification), Unconstr. (Unconstrained), Drv. (Drivers), Neut. (Neutral).

	Adaptive (MEME +)				Neutral / Purifying (MEME -)			
**Dataset**	**Constr. Div.**	**Driven Div.**	**Complex Div.**	**Unconstr. Div.**	**Cryptic Constr.**	**Cryptic Drv.**	**Complex Neut.**	**Non-Sig.**
rbcL (Plant RuBisCO)	47	10	15	11	43	11	41	288
Mammalian Collagen	9	3	7	30	15	5	15	1375
Mammalian VWF	4	0	2	1	30	4	28	323
HIV rt	12	3	4	3	14	7	19	273
Vertebrate Rhodopsin	1	2	0	9	15	8	24	271
Camelid VHH	8	4	8	9	14	2	11	40
Avian REDIC1	0	0	1	45	2	1	1	603
Sperm lysin	10	0	3	13	9	3	7	89
Hepatitis D Ag	3	0	4	12	5	1	5	166
IAV H1N1 HA	4	1	2	17	1	1	1	562
IAV H3N2 HA	2	0	3	12	2	1	4	305
Mammalian RBP3	0	0	1	3	2	3	8	395
Flavivirus NS5	1	0	0	1	7	1	3	329
HIV vif	0	0	0	8	1	1	3	179
ADORA3 (Mammals)	0	0	0	1	4	2	3	97
Mam. beta-globin	0	0	0	0	8	1	1	134
SARS-CoV-2 S	0	0	0	7	2	0	0	1275
Streptococcus PTS	0	0	0	9	0	0	0	630
Drosophila adh	0	0	0	8	0	0	0	246
Mammalian COXI	0	0	0	2	0	1	0	507
SWS1 Opsin (Bats)	0	0	0	3	0	0	0	283
Encephalitis env	0	0	0	1	1	0	0	498
Mammalian AMELX	0	0	0	2	0	0	0	217
Primate Lysozyme	0	0	0	0	0	0	0	130

bioRxiv preprint doi: https://doi.org/10.64898/2026.03.09.710461; this version posted March 11, 2026. The copyright holder for this preprint (which was not certified by peer review) is the author/funder, who has granted bioRxiv a license to display the preprint in perpetuity. It is made available under aCC-BY 4.0 International license.

Table S9: PRIME vs. DMS Validation for Significant H3N2 Sites. Properties shown are individually significant after the full two-stage hierarchical correction ($q < 0.05$, controlling for selective inference across the entire gene). 'DMS Residues' account for 80 percent of the preference mass. A negative Z-score confirms clustering in property space (Constraint). Consistent with Figure 7, sites located in the buried core (e.g., Site 226) exhibit higher importance weights and more restricted experimental variance.

Site	Property	λ	Type	Aln	DMS (WT **bold**)	N_{eff}	Var	Z
186	Polarity (pI)	13.29	Conserved	CGISV	ASN**G**CEMVP	11.3	1.28	-0.95
226	Hydrophobicity	-2.49	Driven	ILQV	TSQVMH**I**FN	13.3	8.46	0.21
	Volume	-1.81	Driven				1026.80	-1.22
	Polarity (pI)	15.00	Conserved				0.91	-1.59
	Helix Prop.	11.82	Conserved				0.05	-1.14
